# Effects of Redox Disturbances on Intestinal Contractile Reactivity in Rats Fed with a Hypercaloric Diet

**DOI:** 10.1155/2018/6364821

**Published:** 2018-10-25

**Authors:** Iara L. L. de Souza, Elba dos S. Ferreira, Anderson F. A. Diniz, Maria Thaynan de L. Carvalho, Fernando R. Queiroga, Lydiane T. Toscano, Alexandre S. Silva, Patrícia M. da Silva, Fabiana de A. Cavalcante, Bagnólia A. da Silva

**Affiliations:** ^1^Programa de Pós-graduação em Produtos Naturais e Sintéticos Bioativos, Centro de Ciências da Saúde, Universidade Federal da Paraíba, João Pessoa, PB, Brazil; ^2^Departamento de Fisiologia e Patologia, Centro de Ciências da Saúde, Universidade Federal da Paraíba, João Pessoa, PB, Brazil; ^3^Centro de Ciências da Saúde, Universidade Federal da Paraíba, João Pessoa, PB, Brazil; ^4^Departamento de Educação Física, Centro de Ciências da Saúde, Universidade Federal da Paraíba, João Pessoa, PB, Brazil; ^5^Programa de Pós-graduação em Biologia Celular e Molecular, Centro de Ciências Exatas e da Natureza, Universidade Federal da Paraíba, João Pessoa, PB, Brazil; ^6^Departamento de Ciências Farmacêuticas, Centro de Ciências da Saúde, Universidade Federal da Paraíba, João Pessoa, PB, Brazil

## Abstract

Few studies have associated the effects of changes in caloric intake and redox disturbances in the gastrointestinal tract. Therefore, the present study aimed at evaluating the hypercaloric diet consumption influence on the contractile reactivity of intestinal smooth muscle, morphology, and oxidative stress of rat ileum. Wistar rats were randomly divided into groups that received a standard diet and fed with a hypercaloric diet for 8 weeks. Animals were euthanized, and the ileum was isolated to isotonic contraction monitoring. Morphology was evaluated by histological staining and oxidative stress by quantification of malondialdehyde levels and total antioxidant activity. Cumulative concentration-response curves to KCl and carbachol were attenuated in rats fed with a hypercaloric diet compared to those that received a standard diet. In addition, an increase in caloric intake promotes a rise in the thickness of the longitudinal smooth muscle layer of rat ileum and tissue malondialdehyde levels, characterizing lipid peroxidation, as well as a decrease in the antioxidant activity. Thus, it was concluded that the consumption of a hypercaloric diet impairs rat intestinal contractility due to mechanisms involving modifications in the intestinal smooth muscle architecture triggered by redox disturbances.

## 1. Introduction

World Health Organization (WHO) defines obesity as a chronic condition characterized by an excessive accumulation of adipose tissue that causes health risk [1]. Therefore, obesity is categorized in the 10th revision of the International Classification of Diseases (ICD-10) at endocrine, nutritional, and metabolic diseases section [2].

Currently, several models develop obesity in animals through genetic mutations. However, most cases of human obesity are considered polygenic because of several gene integration. Thus, when analyzing the genesis of obesity in humans, the induction of this disease in animals through the consumption of highly palatable and hypercaloric diets is indicated as the most appropriate [3].

Recently, our research group established a model of erectile dysfunction in Wistar rat associated to a hypercaloric diet consumption and characterized by an increase in body adiposity, endothelial dysfunction, and systemic oxidative stress [4]. The integral role of oxidative stress in the genesis of diseases affecting smooth muscle cells has been highlighted, mainly due to evidence of free radicals influence on contractility and/or relaxation of smooth muscle cells [5, 6].

The reactive oxygen species (ROS) are signaling agents under physiological conditions and control healing processes, apoptosis, and maintenance of smooth muscle tone and proliferation of this tissue, among others [7, 8]. ROS include a variety of free radicals, such as superoxide anion (O^2−^) and hydroxyl radicals (OH^−^), as well as nonradical oxygen derivatives such as hydrogen peroxide (H_2_O_2_), hypochlorous acid (HClO), peroxynitrite (ONOO^−^), and ozone (O_3_) [9].

An imbalance resulting from overproduction of ROS can damage proteins, lipids, DNA, and other cellular components [10, 11]. In order to contain the formation of these ROS, the organism presents enzymatic and nonenzymatic antioxidant systems, and both play a fundamental role in the prevention of oxidation resulting from ROS [12]. The enzymatic antioxidant system comprises superoxide dismutase (SOD), glutathione peroxidase (GSH-PX) and reductase (GSH-Rd), and catalase, which are the enzymes responsible for removing O^2−^, organic hydroperoxides, and H_2_O_2_, respectively [13, 14]. The nonenzymatic system involves a group of antioxidants that can be complexed in compounds produced *in vivo*, such as glutathione, ubiquinone, and uric acid, and in compounds obtained directly from the diet such as ɑ-tocopherol (vitamin E), *β*-carotene, ascorbic acid (vitamin C), and phenolic compounds such as flavonoids [11, 15].

In view of this information, changes in the balance between oxidative stress and body antioxidant defenses, with a predominance of ROS, observed when there is an increase in caloric intake, raise the probability of the development of organic dysfunctions. However, few studies have reported the effect of a change in dietary pattern on intestinal disorders; despite the abnormalities on intestinal contraction are related to pathophysiological processes, such as constipation, diarrhea, and intestinal colic [16]. Therefore, the aim of this study was to investigate the influence of hypercaloric diet consumption on the contractile reactivity of intestinal smooth muscle, morphology, and oxidative stress on rat ileum.

## 2. Materials and Methods

### 2.1. Animals

Wistar rats (*Rattus norvegicus*), 2 months old (approximately 150 g), were obtained from the Bioterium Professor Thomas George from Universidade Federal da Paraíba (UFPB). The animals were maintained under controlled ventilation and temperature (21 ± 1°C) with water *ad libitum* in a 12 h light-dark cycle (light on from 6 to 18 h). The experimental procedures were performed following the principles of guidelines for the ethical use of animals in applied etiology studies [17] and from the Conselho Nacional de Controle de Experimentação Animal of Brazil [18] and were previously approved by the Ethics Committee on Animal Use of UFPB (protocol no. 0201/14).

### 2.2. Groups and Diets

Animals were randomly divided into two groups (10 rats/group): rats that received a standard diet (Presence®) containing by weight 23% protein, 63% carbohydrate, and 4% lipids with energy density 3.8 kcal/g (SD) and rats fed with a hypercaloric diet composed by a standard diet (Presence®), milk chocolate, peanuts, and sweet biscuit in the proportion of 3 : 2 : 2 : 1 (HD) [19]. The hypercaloric diet containing by weight 23% protein, 45% carbohydrate, and 16% lipids with the energy density of 4.2 kcal/g was prepared weekly and supplied to animals as pellets [4]. The experimental groups were fed for 8 weeks.

### 2.3. Drugs

Potassium chloride (KCl), calcium chloride (CaCl_2_), magnesium chloride (MgCl_2_), sodium chloride (NaCl), and formaldehyde were purchased from Vetec Química Fina Ltda. (Brazil). Sodium bicarbonate (NaHCO_3_) and glucose (C_6_H_12_O_6_) were purchased from Dinâmica (Brazil). Sodium monobasic phosphate (NaH_2_PO_4_), sodium hydroxide (NaOH), and hydrochloric acid (HCl) were purchased from Nuclear (Brazil). These substances, except glucose, NaCl, and NaHCO_3_, were diluted in distilled water to obtain each solution, which was maintained under refrigeration.

Carbamylcholine hydrochloride (CCh) was purchased from Merck (USA). Cremophor®, thiobarbituric acid, tetramethoxypropane, perchloric acid, Mayer's hematoxylin, and eosin were acquired from Sigma-Aldrich (Brazil). All substances were diluted in distilled water as needed for each experimental protocol. The carbogen mixture (95% O_2_ and 5% CO_2_) was obtained from White Martins (Brazil).

### 2.4. Ileum Isolation

Animals were euthanized by guillotine and the ileum was removed, cleaned of connective tissue and fat, immersed in physiological solution at room temperature, and bubbled with carbogen mixture (95% O_2_ and 5% CO_2_). In order to record the isotonic contractions, ileum segments (2–3 cm) were individually suspended in organ baths (5 mL) by cotton yarn and registered on the smoked drum through levers coupled to kymographs (DTF) with a thermostatic pump model Polystat 12002 Cole-Parmer (Vernon Hills) that controlled the organ bath temperature.

The physiological solution of Tyrode was used and has the composition (in mM) as follows: NaCl (150.0), KCl (2.7), CaCl_2_ (1.8), MgCl_2_ (2.0), NaHCO_3_ (12.0), NaH_2_PO_4_ (0.4), and D-glucose (5.5). The pH was adjusted to 7.4, and the ileum was stabilized for 1 h under a resting tension of 1 g at 37°C and bubbled with a carbogen mixture [20].

### 2.5. Contractile Reactivity Measurement

The ileum was assembled as previously described. After a stabilization period of 30 min to verify the organ functionality, a contraction was induced with 30 mM KCl. Subsequently, cumulative concentration-response curves were obtained to KCl (10^−3^−3 × 10^−1^ M) and CCh (10^−9^−3 × 10^−5^ M).

The contractile reactivity was evaluated based on the values of the maximum effect (*E*_max_) and the negative logarithm of the molar concentration of a substance that produced 50% of its maximal effect (pCE_50_) of both contractile agents, calculated from the concentration-response curves obtained. The maximum amplitude obtained from the SD group concentration-response curve was elected as 100%, and the HD was assessed referring to it.

### 2.6. Histological Analysis

Ileum segments were assembled as previously described fixed in 10% formaldehyde solution and subjected to a standard histological procedure. This process was composed of the following steps: (1) tissue dehydration at increasing alcohol series of 70% for 24 h and 80, 96, and 100% (third bath) during 1 h each; (2) tissue diaphanization/bleaching with immersion in 100% xylene alcohol (1 : 1) during 1 h, followed by two baths in pure xylene during 1 h each; (3) tissue embedding in paraffin, wherein the sample was immersed in two baths of liquid paraffin (heated to 50°C) during 1 h each. Then, samples were embedded in new paraffin.

The blocks obtained were cut to 5 *μ*m thick in cross-section of the ileum and stained with Mayer's hematoxylin/eosin [21]. Digital images of histological sections were obtained and analyzed with an optical microscope with an attached camera. In this analysis, two cross-sections per animal were photographed, and the second quadrant of the ileum circumference was used to measure both circular and longitudinal muscle layers using the Leica Qwin 3.1 software [22].

### 2.7. Assessment of Lipidic Peroxidation Levels

Lipid peroxidation was measured by the chromogenic product of 2-thiobarbituric acid (TBA) reaction with malondialdehyde (MDA) that is a product formed as a result of membrane lipid peroxidation [23]. Therefore, ileum segments were homogenized with KCl (1 : 1), and samples of tissue homogenate (250 *μ*L) were incubated at 37°C for 60 min. After that, the mixture was precipitated with 35% perchloric acid and centrifuged at 1207g for 20 min at 4°C. Then, the supernatant was collected and 400 *μ*L of 0.6% TBA was added and incubated at 95–100°C for 1 h. After cooling, the samples were read in a spectrophotometer at a wavelength of 532 nm (Biospectro, SP-220 model-Brazil). The determination of the MDA concentration was made by substituting the absorbance values in the MDA standard curve obtained on the basis of a standard solution (1 *μ*L of 1,1,3,3- tetramethoxypropane in 70 mL distilled water) diluted in series of 250, 500, 750, 1000, 1250, 1500, 1750, 2000, 2250, 2500, 2750, and 3000 *μ*L of distilled water.

### 2.8. Antioxidant Activity Assay

The ileum homogenate was assembled as previously described. In addition, an aliquot of 1.25 mg of DPPH was diluted in ethanol (100 mL), kept under refrigeration, and protected from light. Then, 3.9 mL of DPPH solution was added with 100 *μ*L of the supernatant ileum homogenate on appropriate centrifuge tubes. These tubes were vortexed and left to stand for 30 min, centrifuged at 1207g for 15 min at 20°C. Then, the samples were read in a spectrophotometer at a wavelength of 515 nm (Biospectro, SP-220 model-Brazil) [24].

Results were expressed as the percentage of the oxidation inhibition: AOA = 100 − (((DPPH^·^ R) S/(DPPH^·^ R) W) × 100), where (DPPH^·^ R) S and (DPPH^·^ R) W correspond to the concentration of DPPH^·^ remaining after 30 min, measured in the sample (S) and white (W) prepared with distilled water.

### 2.9. Statistical Analysis

Results were expressed as the mean and standard error of the mean (S.E.M.) and statistically analyzed using Student's *t*-test to the intergroup comparison. Cumulative concentration-response curves were fitted, and pCE_50_ values were obtained by nonlinear regression [25]. Values were significantly different when *p* < 0.05. All data were analyzed by GraphPad Prism® version 5.01 (GraphPad Software Inc., USA), and the visualization of histological sections was performed on Q-Capture® Pro version 7.0 software.

## 3. Results

### 3.1. Contractile Reactivity Measurement

**Figure 1:**
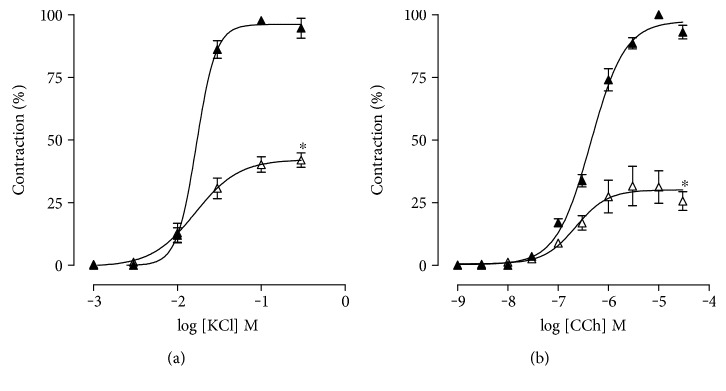
Cumulative concentration-response curves to KCl (a) and CCh (b) in rat ileum from both SD (▲) and HD groups (∆). The symbols and vertical bars represent the mean and S.E.M., respectively (*n* = 5). Student's *t-*test, ^∗^*p* < 0.05 (SD *vs*. HD).

In the HD group, cumulative concentration-response curves to KCl (10^−3^−3 × 10^−1^ M) were attenuated with the reduction on *E*_max_ from 100% (SD) to 42.7 ± 3.1%. However, the pCE_50_ value of the HD group (pCE_50_ = 1.8 ± 0.8) showed no statistical difference compared to the SD group (pCE_50_ = 1.8 ± 0.2) (Figure 1(a), Table 1, *n* = 5).

Meanwhile, cumulative concentration-response curves to CCh (10^−9^−3 × 10^−5^ M) were shifted to the right in rats fed with a hypercaloric diet (pCE_50_ = 6.6 ± 0.1) compared to the SD group (pCE_50_ = 6.3 ± 0.05). In addition, *E*_max_ value was decreased on HD related to SD (*E*_max_ = 32.7 ± 7.5 and 100%, respectively), changing both potency and efficacy of CCh (Figure 1(b), Table 1, *n* = 5).

### 3.2. Histological Analysis

**Figure 2:**
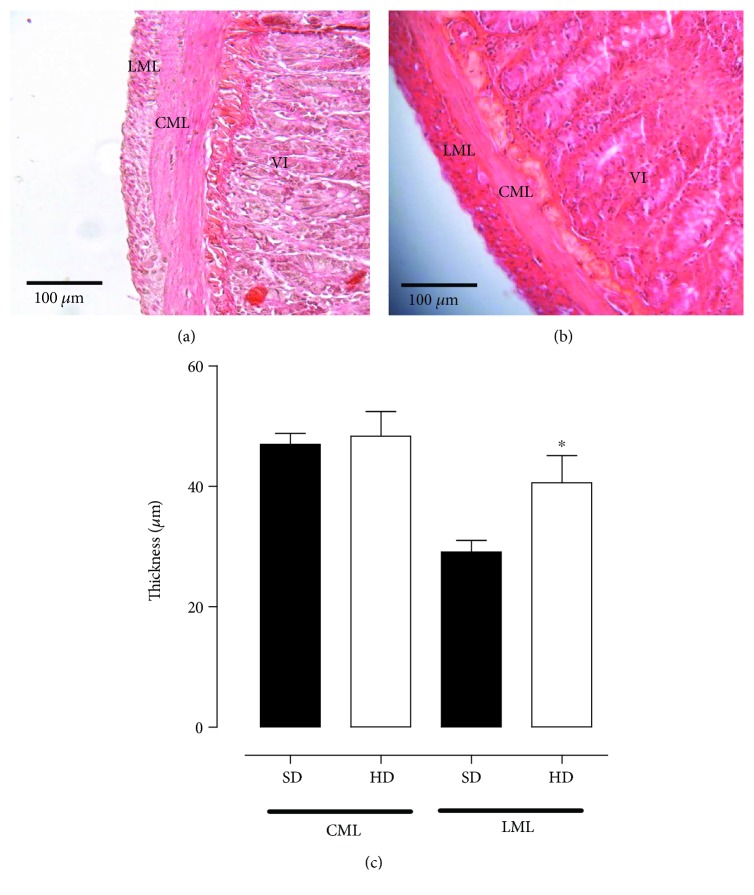
Microphotography of rat ileum from both SD (a) and HD groups (b) and thickness of CML and LML (c). Increased lens 20x. The symbols and vertical bars represent the mean and S.E.M., respectively (*n* = 5). Student's *t-*test, ^∗^*p* < 0.05 (SD *vs*. HD). CML = circular muscle layer; LML = longitudinal muscle layer; VI = villus.

The circular smooth muscle layer thickness of rat ileum has no significant difference between HD (48.3 ± 4.0 *μ*m) and SD groups (47.0 ± 1.8 *μ*m). However, the longitudinal smooth muscle layer of rat ileum presented an increased thickness on rats fed with a hypercaloric diet compared to that in the SD group (36.53 ± 4.6 and 29.0 ± 1.9 *μ*m, respectively) (Figure 2, *n* = 5).

### 3.3. Assessment of Lipidic Peroxidation Levels

**Figure 3:**
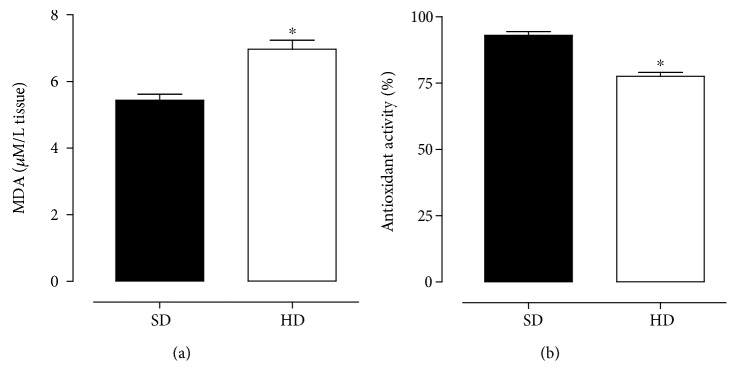
Concentration of MDA (a) and antioxidant activity (b) of rat ileum from both SD and HD groups. The symbols and vertical bars represent the mean and S.E.M., respectively (*n* = 5). Student's *t-*test, ^∗^*p* < 0.05 (SD *vs*. HD). MDA = malondialdehyde.

The MDA levels in rat ileum were increased from 5.4 ± 0.2 *μ*M/L (SD) to 7.0 ± 0.3 *μ*M/L in rats fed with a hypercaloric diet (Figure 3(a), *n* = 5).

### 3.4. Antioxidant Activity Assay

The antioxidant activity in rat ileum was decreased from 93.0 ± 1.4% (SD) to 77.5 ± 1.5% in rats fed with a hypercaloric diet (Figure 3(b), *n* = 5).

## 4. Discussion

In this work, the influence of hypercaloric diet consumption on the contractile reactivity, morphology, and oxidative stress in rat ileum was investigated, demonstrating that an increase in caloric intake is associated with a decrease in contractile reactivity, an increase in the longitudinal smooth muscle layer thickness, lipid peroxidation, and a decrease in the antioxidant activity of this organ.

Chronic noncommunicable diseases (NCDs), such as type 2 diabetes mellitus, dyslipidemias, hypertension, and obesity, play an important and growing role in global public health due to their disabilities and early mortality. In this view, a central part of the genesis of these diseases is the excessive increase in body adiposity [26].

There are many determinants of obesity, being, therefore, a multifactorial disease characterized by the abnormal or excessive accumulation of adipose tissue [1]. Basically, obesity is caused by genetic and environmental factors, which are associated to an imbalance between energy expenditure and caloric consumption that are often determined by the consumption of diets with high energy density and high levels of fat and sugar [27, 28].

The ethical limitation in studying the mechanisms by which obesity induces physiological disorders in humans has resulted in the creation of experimental models using animals that are induced mainly by dietary and/or endocrine manipulation [29]. In these models, it is known that the consumption of hypercaloric/hyperlipidic diets is directly related to the development of various metabolic and hemodynamic disorders that result in adipose tissue hypertrophy/hyperplasia [30, 31].

Nevertheless, few studies have investigated the association of metabolic dysfunctions arising from the consumption of hypercaloric diets with possible alteration of cavernous smooth muscle reactivity on rats. Newly, Wistar rats fed with a hypercaloric diet, during eight weeks, showed increased systemic oxidative stress as well as impairment of contractile and relaxing reactivity of the corpus cavernosum in both pharmaco- and electromechanical couplings [4]. However, there is a lack of information about possible changes in intestinal contractile reactivity due to hypercaloric diet consumption, regarding the caloric content as well as the diet composition.

In view of these premises, it was decided to investigate whether the consumption of this diet, for eight weeks, would also alter the Wistar rats' intestinal contractile reactivity. Thus, the effect of the consumption of hypercaloric diet on both electro- and pharmacomechanical couplings was tested using KCl and CCh, respectively. The KCl was employed to simulate alterations on the membrane potential, which are physiologically controlled by the pacemaker of interstitial cells of Cajal located at the boundaries and in the substance of the inner circular muscle layer from which they spread to the outer longitudinal muscle layer. The CCh was used to mimic the cholinergic stimulation that happens in the intestinal smooth muscle [32, 33].

In this study, the KCl contractile efficacy was reduced in rats fed with hypercaloric diet in relation to those that received standard diet, with no change in potency (Figure 1(a), Table 1). Rembold [34] that verified an attenuation in cumulative concentration-response curves to KCl in rat ileum, decreasing its efficacy without changing the contractile potency, obtained similar results due to exercise. Thus, it is shown that an increase in caloric intake reduces the contraction elicited by the electromechanical coupling of rat ileum.

In addition, when rats consumed hypercaloric diet, a reduction was observed in both contractile efficacy and potency of CCh (Figure 1(b), Table 1). Data obtained by Araujo et al. [35] have demonstrated a similar decrease in both efficacy and potency of CCh in the ileum of rats submitted to acute aerobic swimming exercise that were associated to a possible desensitization of intestinal muscarinic receptors. Moreover, the reperfusion process was also correlated to a reduction of ACh-induced contractile response in the ileum of rats submitted to occlusion of superior mesenteric artery plus interruption of collateral blood flow, leading to reperfusion [36]. Therefore, we demonstrate that an increase in caloric intake reduces the contraction elicited by the pharmacomechanical coupling of rat ileum, due to a less response of smooth muscle cell to cholinergic stimulation.

The synchrony between the smooth muscle layers, a circular and a longitudinal layer, modulates the intestinal contractility. In this view, Bertoni et al. [37] showed that hypertrophy of the circular smooth muscle layer is associated to an increase in contractile efficacy, whereas hypertrophy of the longitudinal smooth muscle layer exhibits a greater sensitivity to the relaxing factors, leading to a decrease in contractile efficacy. Thus, as the present study demonstrated a reduced contractility of rat ileum (Figure 1) due to hypercaloric diet consumption, it was hypothesized that changes in the architecture of the intestinal smooth muscle could be responsible for these results.

Therefore, to verify this hypothesis, histological analyses were performed on rat ileum from both experimental groups. The circular smooth muscle layer thickness was not altered by the consumption of a hypercaloric diet (Figure 2). Interestingly, an increase in the longitudinal smooth muscle layer thickness was observed (Figure 2), characterizing a hypertrophy process. Based on these results, it can be proposed that an increase in caloric intake leads to longitudinal smooth muscle layer hypertrophy and, consequently, reduced the ileum contractility (Figures 1 and 2).

A common problem related to the pathogenesis of intestinal reactivity disorders is the presence of a chronic low-grade inflammation that results in adipose tissue hypertrophy [38]. Similar to other inflammatory processes, adipose tissue inflammation is a trigger for the oxidative stress and can be started by an increase in caloric intake. Briefly, due to the consumption of hypercaloric/hyperlipidic diets, there is an increase in glucose and circulating lipid levels resulting in the excessive supply of energetic substrates to metabolic routes. In turn, ROS production is raised, especially O^2−^, H_2_O_2_, and OH^−^, among others [8, 9].

It is consolidated in the literature that an imbalance in tissue peroxidation and antioxidant activity leads to oxidative damage, consequently, modulating both structure and/or function of the tissue [39–41]. Ischemia-reperfusion events in the intestinal musculature are closely related to oxidative stress [42], promoting motor and intestinal mucosa alteration, decrease in nutrient absorption, and gastrointestinal permeability [43, 44]. Other processes that also alter redox homeostasis, such as physical exercise, have already been correlated to increased lipid peroxidation. Specifically, Araujo et al. [45] showed that chronic aerobic swimming exercise increases lipid peroxidation after four weeks of exercise. Based on this information, it was decided to investigate whether the consumption of a hypercaloric diet would also alter lipid peroxidation of rat ileum. For this, the levels of MDA, a lipid peroxidation marker, were evaluated.

In studies involving oxidative stress, MDA represents a compound formed through the oxidative decomposition of polyunsaturated fatty acids from the membrane and is the most frequently quantified systemic and tissue marker [46]. MDA levels are therefore quantified through a calorimetric reaction in which two molecules of thiobarbituric acid are condensed with a molecule of MDA, and the end product is detected by spectrophotometry technique [47].

According to this methodology, it was observed that MDA concentration was increased in rat ileum from the HD group in relation to the SD group (Figure 3(a)). Souza et al. [4], using the same hypercaloric diet, showed that rats had an increase in MDA levels in plasma, characterizing a systemic oxidative stress. The remarkable increase in the level of ileum peroxidation in the HD group (Figure 3(a)) is quite suggestive that hypercaloric diet consumption also promotes tissue peroxidation. Additionally, it is an important challenge for intestinal redox homeostasis and indicates a possible compromise of the antioxidant defense system of these animals. The peroxidation increase may be a consequence of proinflammatory cytokine production (TNF-*α*, IL-1, and IL-6), due to excess body adiposity, since these cytokines stimulate ROS production by macrophages [48].

In biological systems, this imbalance in ROS production is counterbalanced by the body's antioxidant capacity, representing the body's ability to sequester free radicals through redox systems [49]. Knowing this, it was investigated whether the consumption of a hypercaloric diet would alter the antioxidant activity of these rats. For this, the DPPH reduction colorimetric method was used, which is based on the sample's ability to reduce the DPPH radical (purple) to 1,1-diphenyl 2-picryl hydrazine (translucent), detected by spectrophotometry technique [50].

In this study, the HD group presented a reduction in tissue antioxidant activity in relation to the SD group (Figure 3(b)). The decrease of systemic antioxidant activity was demonstrated by Souza et al. [4] using the same hypercaloric diet. Therefore, the reduction of the ileum antioxidant capacity observed in the HD group (Figure 3(b)) reinforces the idea of an imbalance between ROS production and antioxidant defense systems, correlated with an increase in MDA levels in these rats (Figure 3(a)). Since free radicals are regulators in several cellular processes, such as transcriptional factor activation, gene expression, and cell proliferation [7], it was proposed that an oxidative stress caused by the consumption of a hypercaloric diet may underlie the hypertrophy process of intestinal smooth muscle cells in rats (Figures 2 and 3).

In conclusion, the current study showed initial evidence that the consumption of a hypercaloric diet impairs rat intestinal contractility due to mechanisms involving modifications in the intestinal smooth muscle architecture triggered by redox disturbances. Thus, we provide a model to understand biochemical and metabolic processes involved in the pathophysiological changes caused by the increase in caloric intake, as well as to help to reduce the impact of the various diseases related to it.

## Figures and Tables

**Table 1:** Values of *E*_max_ (%) and pCE_50_ of KCl and CCh in rat ileum from both SD and HD groups. Student's *t-*test, ^∗^*p* < 0.05 (SD *vs*. HD) (*n* = 5).

Groups	KCl		CCh	
	*E*_max_ (%)	pCE_50_	*E*_max_ (%)	pCE_50_
SD	100	1.8 ± 0.2	100	6.3 ± 0.05
HD	42.7 ± 3.1^∗^	1.8 ± 0.8	32.7 ± 7.5^∗^	6.6 ± 0.1^∗^

## Data Availability

The data used to support the findings of this study are available from the corresponding author upon request.
